# The Use of Electricity in the Treatment of Habitual Constipation

**Published:** 1902-12

**Authors:** 


### ABSTRACT.

### The Use of Electricity in the Treatment of Habitual Constipation.

Dr. Sigismund Cohn, of New York, brings forward the claims of electrotherapy in the treatment of habitual constipation, and reports the results of his clinical experiments in this field, in an article published in the *New York Medical Journal*, September 6, 1902. As habitual constipation is an independent disease, due entirely to atony of the muscular coats of the bowels, the chief indication to be met is the restoration of the muscular function of the intestine. Mechanotherapy is generally agreed upon to meet this properly, but massage or gymnastics are usually favored, while electricity is comparatively neglected in the treatment of habitual constipation. The currents used are the galvanic, faradic, and combined faradogalvanic, the sinusoidal and the static. Treatment is started with the static, which the author uses either in the form of the wave current or the static induced current, the latter being especially valuable in the obstinate cases.

Very powerful currents can be used in this way without causing the patient any pain. Both these currents can be made more powerful by a mode of administration called the "swelling current,"meaning a current that starting from zero gradually swells to a maximum point and then recedes, gradually returning to zero. Alternating contractions and relaxations of the muscles are produced by this method. The effect of this mode of stimulation is that the muscles are exercised in a natural way, without any danger of exhausting them. Very good effects were obtained by the author with the use of the sinusoidal current and he prefers it next to the static. The galvanic current he uses only in the form of hydroelectric treatment. Of the sixteen cases reported to illustrate the application of these methods all but three were cured permanently.

Measuring the abdominal circumference and weighing his patients before and after each treatment, he always found a reduction in the abdominal circumference, as well as the weight of the body. The author explains this loss in abdominal circumference by the fact that the powerful contractions of the abdominal muscles, leave these muscles, right after treatment, in a state of improved tonicity (less flabby and more contracted.)

The consequence is that these muscles are able to offer more resistance to the internal abdominal pressure, therefore the capacity of the abdominal cavity will be reduced and the circumference become smaller. The loss of weight is explained by the loss of $H_2O$ and $CO_2$. Electrical and especially static currents produce hyperhydrosis, and the greater elimination of $CO_2$ is explained by the increased work done by the muscles.

